# A Boolean Function for Neural Induction Reveals a Critical Role of Direct Intercellular Interactions in Patterning the Ectoderm of the Ascidian Embryo

**DOI:** 10.1371/journal.pcbi.1004687

**Published:** 2015-12-29

**Authors:** Naoyuki Ohta, Kana Waki, Atsushi Mochizuki, Yutaka Satou

**Affiliations:** 1 Department of Zoology, Graduate School of Science, Kyoto University, Sakyo, Kyoto, Japan; 2 RIKEN Advanced Science Institute, Wako, Saitama, Japan; 3 CREST, Japan Science and Technology Agency, Kawaguchi, Saitama, Japan; New York Medical College, UNITED STATES

## Abstract

A complex system of multiple signaling molecules often produce differential gene expression patterns in animal embryos. In the ascidian embryo, four signaling ligands, Ephrin-A.d (Efna.d), Fgf9/16/20, Admp, and Gdf1/3-r, coordinately induce *Otx* expression in the neural lineage at the 32-cell stage. However, it has not been determined whether differential inputs of all of these signaling pathways are really necessary. It is possible that differential activation of one of these signaling pathways is sufficient and the remaining signaling pathways are activated in all cells at similar levels. To address this question, we developed a parameter-free method for determining a Boolean function for *Otx* expression in the present study. We treated activities of signaling pathways as Boolean values, and we also took all possible patterns of signaling gradients into consideration. We successfully determined a Boolean function that explains *Otx* expression in the animal hemisphere of wild-type and morphant embryos at the 32-cell stage. This Boolean function was not inconsistent with three sensing patterns, which represented whether or not individual cells received sufficient amounts of the signaling molecules. These sensing patterns all indicated that differential expression of *Otx* in the neural lineage is primarily determined by Efna.d, but not by differential inputs of Fgf9/16/20, Admp, and Gdf1/3-r signaling. To confirm this hypothesis experimentally, we simultaneously knocked-down *Admp*, *Gdf1/3-r*, and *Fgf9/16/20*, and treated this triple morphant with recombinant bFGF and BMP4 proteins, which mimic Fgf9/16/20 and Admp/Gdf1/3-r activity, respectively. Although no differential inputs of Admp, Gdf1/3-r and Fgf9/16/20 signaling were expected under this experimental condition, *Otx* was expressed specifically in the neural lineage. Thus, direct cell–cell interactions through Efna.d play a critical role in patterning the ectoderm of the early ascidian embryo.

## Author Summary

It is often difficult to understand a complex system of multiple signaling molecules in animal embryos only with experimental procedures. Although theoretical analysis might solve this problem, it is often difficult to precisely determine parameters for signaling gradients and kinetics of signaling molecules. In the present study, we developed a parameter-free method for determining a Boolean function for understanding a complex signaling system using gene expression patterns of signaling molecules and geometrical configurations of individual cells within the embryo. In the ascidian embryo, four signaling ligands, Ephrin-A.d (Efna.d), Fgf9/16/20, Admp, and Gdf1/3-r, coordinately induce *Otx* expression in the neural lineage at the 32-cell stage. In addition to determining a Boolean function, our method determined sensing patterns, which represented whether or not individual cells received sufficient amounts of the signaling molecules. The sensing patterns predicted that differential expression of *Otx* in the neural lineage is primarily determined by Efna.d, but not by differential inputs of Fgf9/16/20, Admp, and Gdf1/3-r. We confirmed this prediction by an experiment. As a result, we found that only Efna.d signaling pathway is differentially activated between ectodermal cells and the remaining signaling pathways are activated in all ectodermal cells at similar levels.

## Introduction

**Fig 1:**
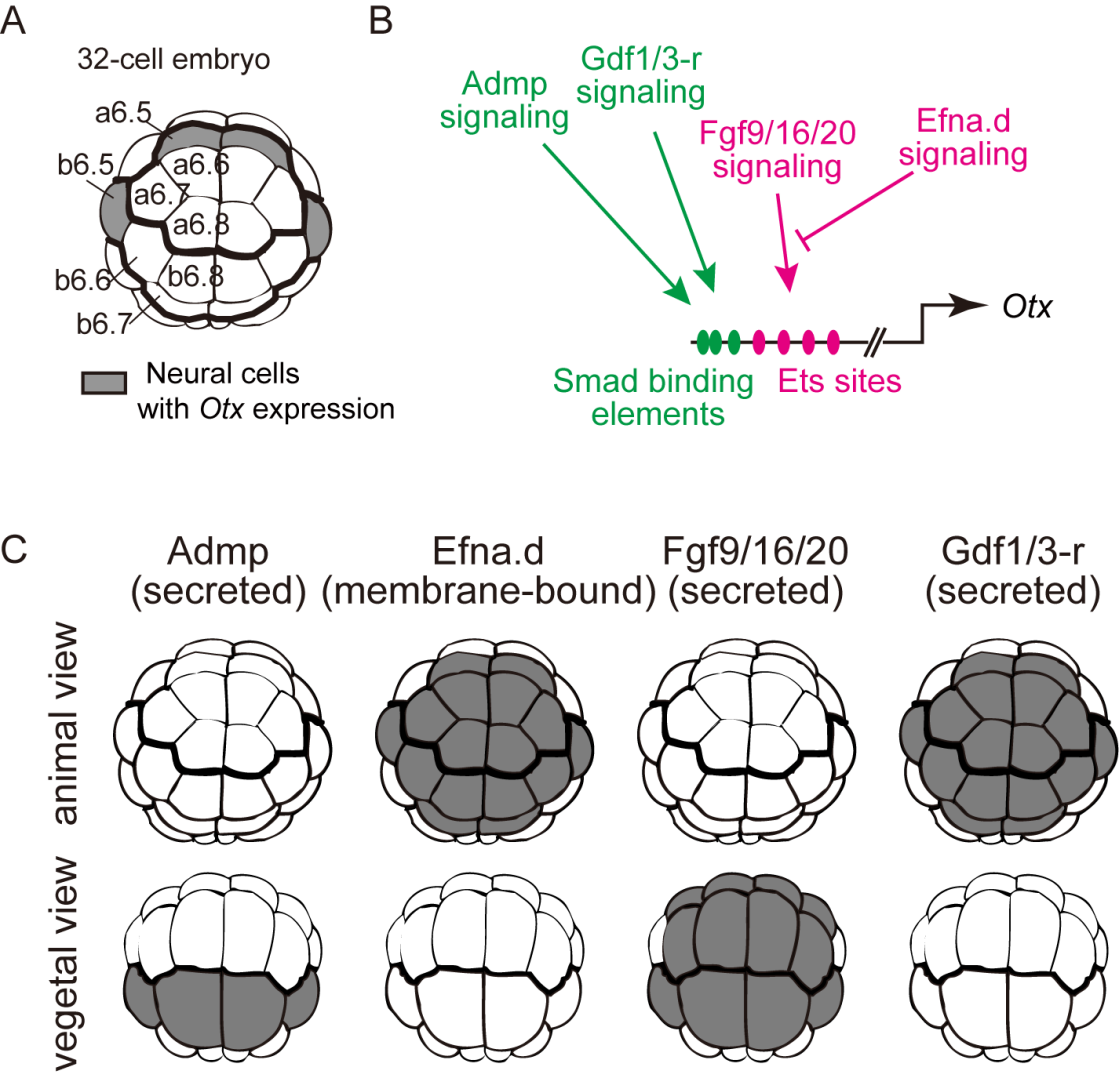
*Otx* expression in the neural lineage of the 32-cell embryo. (A) Schematic illustration of the animal hemisphere of a 32-cell embryo of *Ciona intestinalis*. Blastomere names are indicated in the left half of the bilaterally symmetrical embryo. Although all cells in the animal hemisphere have the potential to express *Otx* upon induction, only the blastomeres colored in gray express *Otx* [2–4]. (B) The specific expression of *Otx* in the neural lineage at the 32-cell stage is controlled by a 200-bp region containing multiple Ets sites and Smad binding elements [2–4]. Fgf9/16/20 signaling positively regulates *Otx* through the Ets binding sites, and Efna.d signaling negatively regulates the Fgf9/16/20 signaling pathway. Admp and Gdf1/3-r negatively regulate *Otx* through the Smad binding elements. (C) Signaling sources of Admp, Efna.d, Fgf9/16/20, and Gdf1/3-r are colored in gray. Given the delay between gene expression and protein translation, we assumed that cells descended from cells expressing a ligand gene at the 16-cell stage would express the encoded protein at the 32-cell stage [3, 4].

In animal embryos, cell-cell interactions directed by secreted and membrane-bound signaling ligands play an important role in establishing specific gene expression patterns. There are 16 ectodermal cells in the animal hemisphere of the 32-cell embryo of the ascidian, *Ciona intestinalis*, and all 16 have the potential to express *Otx* upon induction (Fig 1A). Four signaling molecules, Fgf9/16/20, Admp (anti-dorsalizing morphogenetic protein; a signaling molecule belonging to the BMP subfamily in the TGFβ superfamily), Gdf1/3-r [formerly called Gdf1/3-like and renamed according to the nomenclature guideline recently published [1]], and Efna.d (formerly EphrinA-d), cooperatively regulate *Otx* expression in four cells, which give rise to neural cells [2–4]. Fgf9/16/20 activates *Otx* expression through the ERK pathway, which is antagonized by Efna.d [5, 6]. Admp and Gdf1/3-r negatively regulate *Otx* expression by inducing the binding of the effector transcription factor Smad to an *Otx* enhancer (Fig 1B and 1C). The observation that *Otx* expression expands throughout the ectoderm upon knockdown of *Efna*.*d* or double-knockdown of *Admp* and *Gdf1/3-r* [4] indicates that these three genes are essential for differential expression of *Otx* within the ectodermal cells and patterning the ectoderm. On the other hand, another study indicated that a differential input of Fgf9/16/20 signaling could direct differential *Otx* expression in the ectoderm [7]. Thus, it has not yet been established which of these factors is critical for patterning of the ectoderm of normal embryos. In other words, it has not been determined whether differential inputs of all of these signaling pathways are really necessary. For instance, it is possible that differential activation of one of these signaling pathways is sufficient and the remaining signaling pathways are activated in all cells at similar levels. Because our previous experiments [4] did not necessarily give an answer to this question, we took an advantage of theoretical analysis in the present study.

Although quantitative models have successfully simulated molecular gradients for embryonic patterning in other model systems [8–10], it is difficult to precisely determine parameters for signaling gradients and kinetics of signaling molecules in the ascidian embryo. Boolean functions provide an alternative, because inputs and outputs are treated as binary values, and parameters that are difficult to determine are not used. In previous studies, Boolean functions have successfully explained how combinations of different transcription factors determine specific gene expression patterns [11–15]. Here we report determination of a Boolean function for *Otx* expression in the 32-cell embryo of *Ciona intestinalis*. This function reveals how individual cells sense signaling inputs and which signaling is the limiting factor for patterning the ectoderm.

## Results

### Determination of a Boolean function in a hypothetical biological system

**Fig 2:**
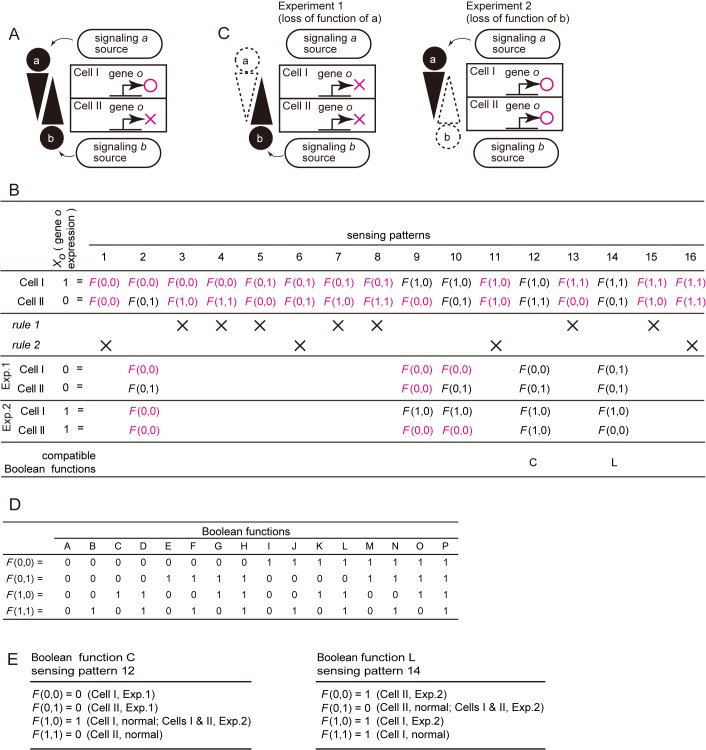
Boolean functions in a hypothetical biological system. (A) A hypothetical biological system, consisting of two initially equivalent cells I and II, and two signaling molecules *a* and *b*. After a sufficient period of time, gene *o* is expressed only in cell I but not in cell II. (B) A Boolean function that describes expression of gene *o* is represented by *X*_*o =*_ *F*(*X*_*a*_, *X*_*b*_), where *X*_*a*_ and *X*_*b*_ represent states of signaling pathways downstream of signaling molecules *a* and *b* (1, active; 0, inactive; collectively called “sensing patterns”), and *X*_*o*_ represents expression of gene *o*. The 16 logically possible sensing patterns are shown in the second row. Sensing patterns and Boolean functions incompatible with *Rules 1* and *2* (see text) are indicated by ‘X’ in the third row. The fourth and fifth rows show sensing patterns appearing in Experiments 1 and 2, which are shown in (C). Sensing patterns incompatible with *Rules 1* and *2* are shown in magenta in the second, fourth and fifth rows. The sixth row shows a Boolean function compatible with the sensing pattern indicated in each column among all of the logically possible Boolean functions shown in (D). (C) Two conceptual loss-of-function experiments. (D) Sixteen Boolean functions that are logically possible for two input variables. (E) Two distinct combinations of Boolean functions and sensing patterns that explain the expression of gene *o* in this hypothetical system.

Here we introduce a method for determining a Boolean function of gene expression directed by extracellular signals within a population of equivalent cells. Before formalizing neural induction of the ascidian embryo, we first considered a Boolean function describing a simple hypothetical biological system illustrated in Fig 2A. This system consists of two cells, I and II, and two signaling molecules, *a* and *b*. Cells I and II initially express the same set of transcription factors, and are therefore equivalent. After a sufficient period of time, gene *o* is activated only in cell I but not in cell II under control of signaling molecules *a* and/or *b*. Hence, the Boolean function for the expression of gene *o* is represented by [*X*_*o =*_ *F*(*X*_*a*_,*X*_*b*_)], where *X*_*o*_ represents expression of gene *o*, and *X*_*a*_ and *X*_*b*_ represent the signaling states of *a* and *b*. Inputs and outputs are considered in binary space. If a signal sufficiently activates its intracellular pathway, it is represented as ‘1’, and otherwise as ‘0’ hereafter. Because there are 4 (= 2^2^) possible combinations of input signaling states in each cell in this hypothetical system, there are 16 (= 4×4) possible states in the whole system (Fig 2B). These states, which are represented as (*X*_*a*_,*X*_*b*_), are called "sensing patterns" hereafter, because they represent how individual cells sense individual signaling inputs. In this hypothetical system, signaling molecule *a* comes from the upper side of Fig 2A, and signaling molecule *b* comes from the lower side. Obviously, eleven sensing patterns are incompatible with the following two simple principles, which we call *Rule 1* and *Rule 2*.

#### Rule 1: Signaling sources

Because signal *a* comes from the upper side of Fig 2A, the strength of signaling *a* should not be weaker in cell I than in cell II. Namely, whenever the signaling state of *a* in cell II is ‘1’, the signaling state of *a* in cell I is always ‘1’. For this reason, among the 16 sensing patterns shown in Fig 2B, sensing patterns 3, 4, 7 and 8 cannot stand (Fig 2B). Similarly, the strength of signaling *b* should not be weaker in Cell II than in Cell I; therefore, sensing patterns 5, 7, 13 and 15 cannot stand.

In other words, according to *Rule 1*, there are three possible combinations of the signaling states of *a*, (cell I = 1, cell II = 1), (cell I = 1, cell II = 0), and (cell I = 0, cell II = 0). Similarly, there are three possible combinations of the signaling states of *b*. These numbers are calculated as [(the number of cells) + 1] (formula *1*); in this hypothetical system, 2 + 1 = 3. Because we consider two signaling molecules in the hypothetical system, there are 9 (= 3^2^) combinations of the signaling states, as shown in Fig 2B; sensing patterns 1, 2, 6, 9, 10, 11, 12, 14, and 16.

#### Rule 2: Uniqueness

Signaling states should be different between cell I and cell II [15–17]. Sensing patterns 1, 6, 11 and 16 do not satisfy this rule, because the expression of gene *o* is not uniquely determined. Hence, these four patterns cannot stand (Fig 2B).

Next, we introduce hypothetical loss-of-function of signaling molecules *a* and *b* (Fig 2C), in which loss-of-function of signaling molecule *a* results in loss of expression of gene *o* (Experiment 1), and loss-of-function of signaling molecule *b* induces ectopic expression of gene *o* in cell II (Experiment 2). If we assume sensing pattern 2, Experiment 1 does not alter the sensing pattern but the expression of *o* is lost in cell I. Hence, sensing pattern 2 is not consistent with *Rule 2* under this experimental condition. In other words, the assumption of sensing pattern 2 necessitates *F*(0,0) = 1 and *F*(0,0) = 0 simultaneously, and therefore this assumption cannot stand. Similarly, sensing patterns 9 and 10 cannot stand, leaving only sensing patterns 12 and 14.

Fig 2D shows the 16 possible Boolean functions with two binary input values and one binary output value (Boolean functions A to P). Among them, only Boolean function C is compatible with sensing pattern 12, and only Boolean function L is compatible with sensing pattern 14 (Fig 2E).

Our method raises two important points. First, although the results of the above hypothetical experiments might lead to the intuitive interpretation that *a* activates *o* and *b* represses *o*, the logical considerations enumerated above indicate that these two hypothetical experiments cannot distinguish the two possibilities shown in Fig 2E. Thus, our method gives a precise and strict interpretation for the experimental results.

Second, Fig 2E predicts that these two possibilities can be distinguished by simultaneous loss-of-function or gain-of-function of *a* and *b*, because outputs for *F*(0, 0) and *F*(1, 1) are predicted to be different between these two possibilities. Thus, this method can provide a practical direction of experiments.

We also consider a similar case, in which signaling molecule *b* is tethered to the plasma-membrane of the signaling source, and therefore only cell II but not cell I can receive *b*, as shown in S1A Fig. In this case, sensing patterns, 5, 6, 7, 8, 13, 14, 15, and 16, are ruled out on the basis of the assumption that cell I cannot receive *b*. By considering this case in the same way as in Fig 2, only a sensing pattern 12, which is compatible with Boolean function C, stands (S1B–S1D Fig).

Finally, we consider another case, in which membrane-tethered signaling molecule *b* is expressed additionally in cell I and cell II, as shown in S2A Fig. Because cell I receive *b* only from cell II and cell II receive *b* from both of cell I and the signaling source, the strength of signaling *b* should not be weaker in cell II than in cell I. Namely, whenever the signaling state of *b* in cell I is ‘1’, the signaling state of *b* in cell II is always ‘1’. This assumption is the same as the one we consider in the first hypothetical system shown in Fig 2. Therefore, as we showed in the first hypothetical system, two sensing patterns 12 and 14 stand (S2A–S2D Fig). Namely, our method can treat both freely diffusible molecules and membrane-tethered molecules with a small modification.

### A Boolean function for *Otx* expression in the neural lineage at the mid-to-late 32-cell stage

We next applied this logic to the signaling system that induces the neural marker gene *Otx* in the neural lineage (a6.5 and b6.5) of the 32-cell *Ciona* embryos (Fig 1A). Previous studies revealed that four signaling molecules, Admp, Efna.d, Fgf9/16/20 and Gdf1/3-r, are directly involved in inducing *Otx* expression in two pairs of cells (a6.5 and b6.5) within 16 equivalent ectodermal cells (eight pairs of cells) in the animal hemisphere (Fig 1A–1C) [2–4]. The activities of these signaling pathways are denoted by the binary variables, *X*_*admp*_, *X*_*efn*_, *X*_*fgf*_, and *X*_*gdf*_, and the expression of *Otx* (*X*_*otx*_) is represented by a Boolean function, *X*_*otx =*_ *F*(*X*_*admp*_, *X*_*efn*_, *X*_*fgf*_, *X*_*gdf*_).

In this biological system, there are four binary input variables and eight pairs of equivalent cells, and the number of possible sensing patterns is 4,294,967,296 [= (2^4^)^8^]. As in the case of the hypothetical biological systems, we first screened individual sensing patterns with *Rule 1* and *Rule 2*.

#### Rule 1

On the basis of the expression patterns of *Admp*, *Efna*.*d*, *Fgf9/16/20* and *Gdf1/3-r* (Fig 1C) [3, 18], and the three-dimensional geometrical relationships among blastomeres of the 32-cell embryo [7], we inferred relative signaling strength among blastomeres for four signaling molecules under an assumption that differences in the area of contact of cells with cells expressing signaling ligands are correlated with signaling strength.

This assumption is based on a previous study showing that differences in the area of contact between competent cells and inducing cells are associated with selecting the induced cells in induction by Fgf9/16/20 [7]. For example, if a cell has a smaller area of contact with cells expressing an inducing molecule than another cell, we assume that the signaling strength is not stronger in the first cell than in the second cell. Because the anterior cells (a-line cells) did not directly contact cells that express Admp, we assumed that Admp signaling would not be weaker in a cell closer to the signaling source than in a cell farther from the signaling source. For the calculation, we ruled out autocrine effects of Efna.d, because it is a GPI-anchored membrane protein. We considered that the other three secreted molecules can act in both autocrine and paracrine manner. Because Efna.d is expressed in the entire animal hemisphere, we treated this molecule on the basis of the consideration in S2 Fig in the subsequent analysis.

**Fig 3:**
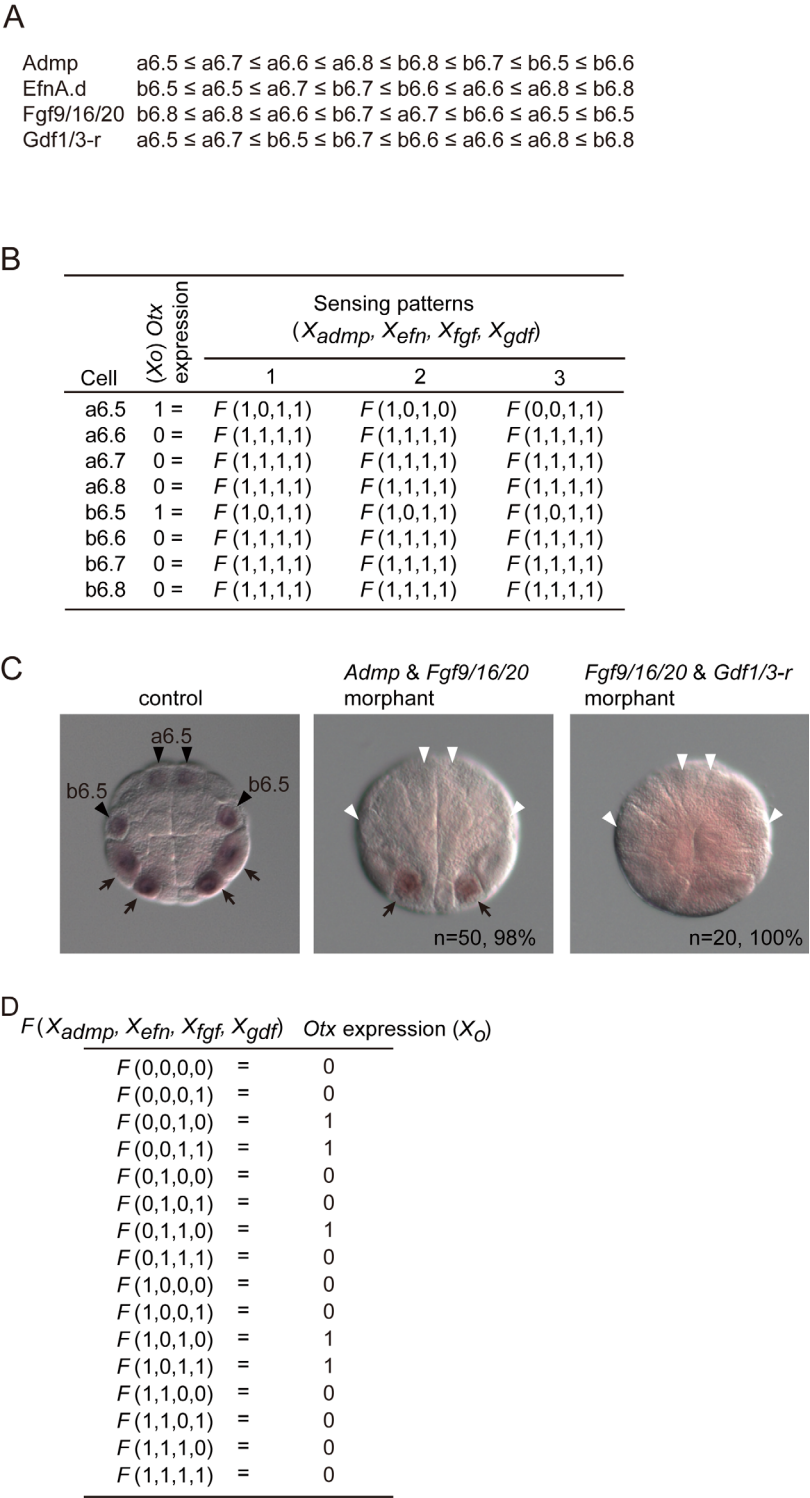
The Boolean function that explains neural induction of the ascidian 32-cell embryo. (A) Estimated order of signaling strength of the Admp, Efna.d, Fgf9/16/20 and Gdf1/3-r pathways in the animal hemisphere, from which ectodermal cells are derived, of a mid-to-late 32-cell embryo. This estimation is based on the data shown in S1 Table. (B) Sensing patterns for Admp, Efna.d, Fgf9/16/20 and Gdf1/3-r signaling that are not inconsistent with *Otx* expression in normal and experimental embryos (S2 Table). These sensing patterns are compatible with 16 or 8 Boolean functions shown in S2 Fig. (C) *Otx* expression in a control, a morphant for *Admp* and *Fgf9/16/20*, and a morphant for *Fgf9/16/20* and *Gdf1/3-r*. Black arrowheads indicate the expression of *Otx* in the neural lineage (a6.5 and b6.5 blastomeres). White arrowheads indicate that the expression of *Otx* in the neural lineage was lost. Arrows indicate *Otx* expression in the vegetal hemisphere. (D) Boolean function to direct *Otx* expression.

Because the mid-to-late 32-cell period is crucial for induction of *Otx* through the MEK pathway [7], we used geometrical data for a cell from this stage to calculate the contact area with surrounding cells expressing signaling ligands (S1 Table). The inferred order of cells for the signaling strength of each of the above signaling molecules is illustrated in Fig 3A as an inequality. According to *Rule 1*, signaling states for each of the four signals within the eight pairs of cells need to satisfy the inequality. Therefore, the number of possible sensing patterns for each of the four signals within the eight pairs of cells is nine (= 8+1), according to formula *1*. In other words, the number of possible sensing patterns was reduced to 6,561 (= 9^4^).

#### Rule 2

Because *Otx* is expressed only in a6.5 and b6.5, sensing patterns in a6.5 and b6.5 cells should not be the same as those of the remaining six pairs of cells. In addition, we used data from previous studies in which *Admp*, *Efna*.*d*, *Fgf9/16/20* and *Gdf1/3-r* genes were knocked down and overexpressed [4] (The data set was summarized in S2 Table). After the 6,561 sensing patterns described above were screened with *Rule 2*, three sensing patterns remained (sensing patterns 1 to 3; Fig 3B). These three sensing patterns commonly indicate that Fgf9/16/20 signaling is active in all cells, and Efna.d signaling is inactive in the neural lineage, while the signaling states of Admp and Gdf1/3-r were not uniquely determined. That is, from the results in the previous reports [3, 4], we cannot discriminate possibilities that Admp and Gdf1/3-r signaling may or may not be involved in patterning the ectoderm.

The Boolean function was not determined uniquely, either. The above three sensing patterns, 1, 2, and 3, were compatible with 16, 8, and 8 redundant Boolean functions, respectively (S3 Fig), because output values for *F*(*X*_*admp =*_ 0, *X*_*efn =*_ 0, *X*_*fgf =*_ 0, *X*_*gdf =*_ 1), *F*(*X*_*admp =*_ 0, *X*_*efn =*_ 1, *X*_*fgf =*_ 0, *X*_*gdf =*_ 1), *F*(*X*_*admp =*_ 1, *X*_*efn =*_ 0, *X*_*fgf =*_ 0, *X*_*gdf =*_ 0), and *F*(*X*_*admp =*_ 1, *X*_*efn =*_ 1, *X*_*fgf =*_ 0, *X*_*gdf =*_ 0) were not determined. To determine output values for these functions, we examined *Otx* expression in two double morphants: *Admp* and *Fgf9/16/20*, and *Fgf9/16/20* and *Gdf1/3-r*. *Otx* expression in the animal hemisphere was lost in these morphants (Fig 3C). By applying *Rule 2* on these results, we found that three sensing patterns were compatible with only one Boolean function (Fig 3D; function A in S3 Fig), while these sensing patterns were still remained.

These three sensing patterns all indicated that Efna.d is a critical factor for patterning the ectoderm, for the following reason. In the case of sensing pattern 1, the critical role of Efna.d was obvious, because signaling of the other three molecules was sufficiently active in all cells. In the case of sensing pattern 2, Gdf1/3-r signal was not sufficiently active in a6.5. However, the Boolean function indicated that Gdf1/3-r input was not important for *Otx* expression in a6.5, because both of *F*(*X*_*admp =*_ 1, *X*_*efn =*_ 0, *X*_*fgf =*_ 1, *X*_*gdf =*_ 0) and *F*(*X*_*admp =*_ 1, *X*_*efn =*_ 0, *X*_*fgf =*_ 1, *X*_*gdf =*_ 1) gave the same result of *X*_*o =*_ *1* (Fig 3D). On the other hand, *F*(*X*_*admp =*_ 1, *X*_*efn =*_ 0, *X*_*fgf =*_ 1, *X*_*gdf =*_ 0) and *F*(*X*_*admp =*_ 1, *X*_*efn =*_ 1, *X*_*fgf =*_ 1, *X*_*gdf =*_ 0) gave different outputs of *X*_*o =*_ *1* and *X*_*o =*_ *0*, respectively. Hence, Efna.d signaling was the only candidate that could pattern the ectoderm. Similarly, in the case of sensing pattern 3, although Admp signal was not sufficiently active in a6.5, this was not important for *Otx* expression; both of *F*(*X*_*admp =*_ 0, *X*_*efn =*_ 0, *X*_*fgf =*_ 1, *X*_*gdf =*_ 1) and *F*(*X*_*admp =*_ 1, *X*_*efn =*_ 0, *X*_*fgf =*_ 1, *X*_*gdf =*_ 1) gave the same result of *X*_*o =*_ *1* (Fig 3D). Thus, the three sensing patterns described above all indicated that a differential input of Efna.d is critical for patterning the ectoderm.

### Boolean functions for *Otx* expression in the neural lineage at the early 32-cell stage

During the 32-cell stage, the *Ciona* embryo dynamically changes its shape. Therefore we tried to determine sensing patterns and Boolean functions for three early 32-cell embryos, for which geometric data were obtained in a previous study [7] (S3 Table; S4A–S4C Fig). In these analyses, only geometric data were different from the first analysis for the mid-to-late 32-cell embryo. We obtained the same four sensing patterns from each of these three virtual embryos (S4D Fig). Three of them were the same as the ones obtained from the mid-to-late 32-cell embryo, and were compatible with the same Boolean function as that obtained from the mid-to-late 32-cell embryo. The remaining one, sensing pattern 4, was slightly different, and was compatible with eight Boolean functions, one of which was the Boolean function compatible with the other three sensing patterns (S4D and S4E Fig).

Even under sensing pattern 4, the Boolean functions compatible with this sensing pattern indicated that Efna.d is a critical factor for patterning the ectoderm. Sensing pattern 4 showed that Efna.d signaling and Gdf1/3-r signaling are not sufficiently active in a6.5 and b6.5, implying that these two factors are candidates for a factor for patterning the ectoderm. However, all eight Boolean functions compatible with sensing pattern 4 indicated that Gdf1/3-r signaling cannot pattern the ectoderm under this sensing pattern, because *F*(*X*_*admp =*_ 1, *X*_*efn =*_ 0, *X*_*fgf =*_ 1, *X*_*gdf =*_ 0) and *F*(*X*_*admp =*_ 1, *X*_*efn =*_ 0, *X*_*fgf =*_ 1, *X*_*gdf =*_ 1) gave the same result, *X*_*o =*_ *1*. Therefore, even if *Otx* expression in early 32-cell embryos is directed by a Boolean function different from the one in the mid-to-late 32-cell embryo, our analysis indicated that Efna.d is the limiting factor for patterning the ectoderm of the 32-cell embryo.

### Patterning of the ectoderm by Efna.d

**Fig 4:**
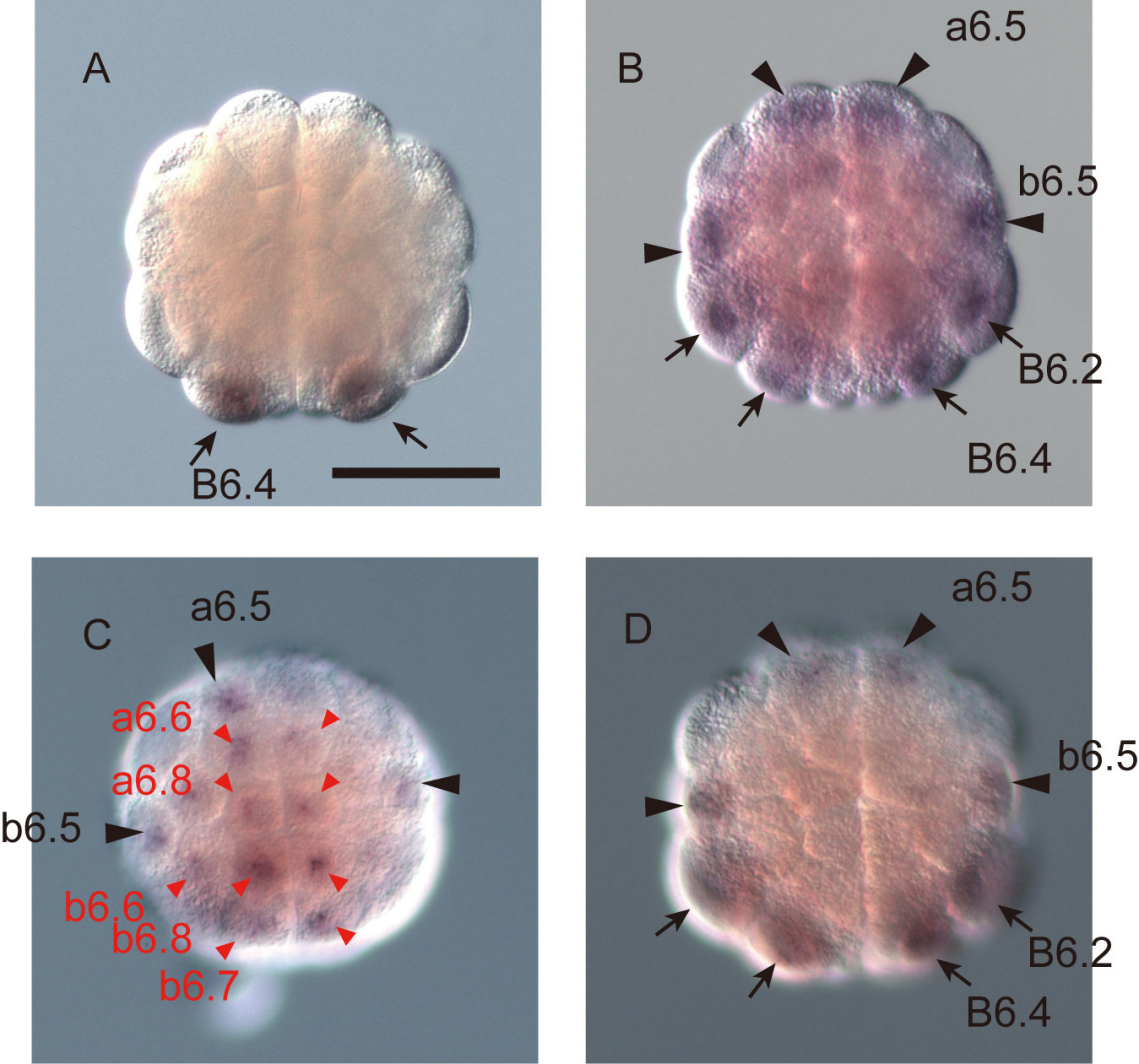
Efna.d signaling plays the key role in patterning of the ectoderm. (A) *Otx* is not expressed in a morphant for *Fgf9/16/20*, *Admp*, and *Gdf1/3-r*, as previously reported [4]. (B) *Otx* is specifically expressed in the neural lineage of morphants for *Fgf9/16/20*, *Admp*, and *Gdf1/3-r* incubated with 1 ng/mL human bFGF and 100 ng/mL human BMP4, although no differential inputs of Fgf9/16/20, Admp and Gdf1/3-r are expected. (C) *Otx* expression is observed not only in the neural lineage but also in the epidermal lineage of morphants for *Fgf9/16/20*, *Admp*, *Gdf1/3-r*, and *Efna*.*d* incubated with bFGF and BMP4. (D) *Otx* is expressed specifically in the neural lineage of embryos injected with a control morpholino oligonucleotide, which does not have any target mRNAs encoded in the *Ciona* genome. We injected 15 fmole of each of morpholino oligonucleotides against *Fgf9/16/20*, *Admp*, *Gdf1/3-r*, and *Efna*.*d* in (A-C), and 60 fmole of the control morpholino oligonucleotide in (D). Black and red arrowheads indicate the expression of *Otx* in the neural and epidermal lineages, respectively. Arrows indicate the expression in the vegetal cells.

The above prediction that Efna.d is the limiting factor for patterning the ectoderm of the 32-cell embryo was consistent with our observation in a previous study that *Otx* expression is expanded throughout epidermal cells of Efna.d morphants [4]. However, it has not been determined whether differential inputs of Fgf9/16/20, Admp, and Gdf1/3-r are really unnecessary for patterning the ectoderm. To test this, we used triple morphants of *Fgf9/16/20*, *Admp*, and *Gdf1/3-r*. First we confirmed our previously result that *Fgf9/16/20*/*Admp*/*Gdf1/3-r* morphants do not express *Otx* in the animal hemisphere [4] (Fig 4A). Next, we incubated *Fgf9/16/20/Admp/Gdf1/3-r* morphants in sea water containing recombinant bFGF and BMP4 proteins, which mimic Fgf9/16/20 and Admp/Gdf1/3-r activity [4]. The bFGF concentration was determined empirically on the basis of our previous study [4]; *Otx* is expressed on average in two cells of *Fgf9/16/20* morphants incubated with 1 ng/mL of bFGF [4]. In this experimental condition, in which no gradients of bFGF and BMP4 within embryos were expected, *Otx* was expressed predominantly in the neural lineage, as in control embryos (Fig 4B; Table 1). Although relatively weak *Otx* expression in the epidermal lineage was observed only in a very small number of embryos, cells with neural fate almost always expressed *Otx* in these embryos, and ectopic expression was also observed in one unperturbed embryo (Table 1). In addition, as expected in our previous study [4], *Otx* expression was observed in the neural and epidermal lineages of quadruple morphants of *Fgf9/16/20*, *Admp*, *Gdf1/3-r*, and *Efna*.*d* incubated in sea water containing recombinant bFGF and BMP4 proteins (Fig 4C; Table 1), while injection of the same amount of a control morpholino oligonucleotide did not affect *Otx* expression (Fig 4D; Table 1). Thus, as predicted by the theoretical model, a difference in strength of Efna.d signaling, which is known to attenuate ERK activation [5, 6], can evoke specific *Otx* expression without differential inputs of Fgf9/16/20, Admp and Gdf1/3-r, even if differential inputs of these factors might contribute to specific *Otx* expression in normal embryos.

**Table 1:** Numbers of embryos that expressed *Otx* under three different conditions.

Expression*	unperturbed control	Control morpholino oligonucleotide	*Fgf9/16/20*/*Admp*/*Gdf1/3-r* morphants	*Fgf9/16/20*/*Admp*/*Gdf1/3-r* morphants incubated with bFGF + BMP4	*Fgf9/16/20*/*Admp*/*Gdf1/3-r*/*Efna*.*d* morphants incubated with bFGF + BMP4
Only in a6.5 and/or b6.5	49	35	1	18	0
a6.5/b6.5 and ectopic in the animal hemisphere	1	0	0	3	20
Ectopic only	0	0	0	0	12
No expression	14	22	27	14	6

* Because some control embryos express *Otx* in a6.7, and this *Otx* expression in a6.7 is not tightly regulated [4, 7], *Otx* expression in a6.7 is not included in this Table.

## Discussion

We determined a Boolean function for *Otx* expression in the animal hemisphere of the mid-to-late 32-cell ascidian embryo, based on a theoretical analysis using data obtained in previous studies [2–4, 7, 18] and in this study. We found that three sensing patterns of signals are compatible with this Boolean function. It is possible that the 32-cell-embryo normally takes only one of these sensing patterns. However, because the Boolean function indicates that *Otx* is specifically expressed in the neural lineage under either of these sensing patterns, the choice of sensing patterns by 32-cell embryos might not be strictly determined. In other words, these sensing patterns might represent fluctuations of signaling and robustness of this system.

The cis-regulatory module of *Otx* for the expression in the neural lineage contains multiple Ets-binding sites and Smad-binding elements (SBEs) [3, 4] (Fig 1B). Ets is positively regulated by Fgf9/16/20 signaling and negatively regulated by Efna.d signaling. SBEs are responsive to signaling of Admp and Gdf1/3-r, and negatively regulate the expression of *Otx* in the neural lineage. The Boolean function in Fig 3D indicates that Fgf9/16/20 and Efna.d work positively and negatively. It also indicates that Admp and Gdf1/3-r have a redundant function, because these two molecules are interchangeable. Thus, although no particular cis-regulatory mechanism was assumed in the present study, the cis-regulatory module is not inconsistent with the Boolean function that we revealed in the present study.

Our theoretical method does not use quantitative parameters that cannot be easily measured, such as the kinetics of individual signaling molecules. Instead, we only use expression patterns of signaling molecules and geometrical configurations of individual cells within the embryo. The former was determined by *in situ* hybridization [3, 18], and the latter was determined by computation of a series of confocal images [7]. Although activities of signaling pathways were treated as binary values, gradients or differential inputs of signaling molecules were taken into consideration. For this purpose, we assumed that the area of the contact of a cell with its surrounding cells that express a ligand is correlated with the strength of signaling. This is the case at least for Fgf9/16/20 [7], and it will be hard to imagine cases in which this assumption is inappropriate in early ascidian embryos with the following two reasons. First, our assumption also takes into consideration a case in which diffusion is very fast and no gradient is formed. Second, if an antagonist altered the activity of a signaling molecule within the embryo, this molecule could be considered as an additional signaling molecule. However, no genes for known antagonists for Fgf9/16/20, Admp, and Gdf1/3-r are expressed from the zygotic genome at or before the 32-cell stage [18].

The sensing patterns of individual cells in normal embryos showed that cells that do not sense Efna.d signaling above a threshold level give rise to neural cells, whereas cells that sense sufficient levels of Efna.d signaling give rise to epidermal cells. A previous study indicated that a differential input of Fgf signaling can differentiate ectodermal cells to neural cells under some experimental conditions and Fgf signaling is thought to be transmitted stronger in neural cells [7]. The present study does not necessarily rule out a possibility that a differential input of Fgf signaling contributes to patterning of the ectoderm in a normal embryo. A differential input of Fgf signaling will indeed contribute to patterning of the ectoderm in a normal embryo with the following three reasons: (1) Fgf signaling might be stronger in neural cells than in epidermal cells [7] (Fig 3A); (2) Efna.d signaling attenuates the ERK pathway activated by Fgf9/16/20 [5, 6] (Fig 1B); (3) a differential level of activation of the ERK pathway controls the expression of *Otx* [2–4]. However, our results indicate that a differential input of Efna.d is essential for the initial patterning of the ectoderm at the 32-cell stage in a normal embryo.

Secreted molecules often form continuous gradients, which are used for patterning of animal embryos [19]. Our result indicates that concentration gradients of Fgf9/16/20, Admp and Gdf1/3-r, or differential inputs of them, are not required, although these molecules are required for establishing the proper expression pattern of *Otx*. Efna.d is a membrane-bound protein, and therefore cannot form a continuous gradient as secreted molecules do. Cells located near the animal pole are surrounded by ectodermal cells, and are therefore expected to receive a stronger Efna.d signal. On the other hand, cells located in the periphery of the animal hemisphere are not completely surrounded by ectodermal cells, and are therefore expected to receive a weaker Efna.d signal. This is reminiscent of the differentiation of inner cell mass and trophectoderm of mammalian embryos. The fate choice between them mainly depends on Hippo signaling, which is thought to be activated through direct cell–cell interaction as Efna.d signaling [20, 21]. In the early animal embryo, cell–cell interaction through direct contacts may provide a more robust system for creating sharp boundaries of gene expression.

## Materials and Methods

### Contact areas of cells with surrounding cells expressing signal ligands

The contact areas of individual animal blastomeres of the 32-cell embryo with cells expressing Admp, Efna.d, Fgf9/16/20 and Gdf1/3-r were calculated using four different 3D-virtual embryos, which were reconstructed virtually from several series of confocal images [7] and the expression patterns of these genes [3, 18]. Given the delay between gene expression and protein translation, we assumed that cells descended from cells expressing a ligand gene at the 16-cell stage would express the encoded protein at the 32-cell stage [4]. The contact surfaces of individual animal blastomeres of the 32-cell embryo with anterior vegetal cells expressing Fgf9/16/20 were previously calculated [7]. We recalculated the contact surfaces of individual animal blastomeres of the 32-cell embryo with all cells expressing Fgf9/16/20 using geometrical data [7], in our previous study [4] and the present study. The contact surfaces of individual animal blastomeres with cells expressing Efna.d for one early 32-cell embryo were also calculated previously [4]. In the present study, we calculated the contact areas of individual cells with cells expressing Admp and Gdf1/3-r (S1 and S3 Tables) for three early 32-cell embryos and one mid-to-late 32-cell embryo. The files we used were downloaded from the Aniseed database [22] (http://www.aniseed.cnrs.fr), and the file names are shown in S1 and S3 Tables. Note that we ruled out autocrine effects of Efna.d, because it is a GPI-anchored membrane protein.

### Gene knockdown and whole-mount in situ hybridization

*C*. *intestinalis* (type A) adults were obtained from the National Bio-Resource Project for *Ciona*. The morpholino oligonucleotides for *Fgf9/16/20*, *Admp*, *Gdf1/3-r*, *and Efna*.*d* used in this study were those used in our previous study [4]. These morpholino oligonucleotides were designed to block translation. We also used a standard control MO (5’-CCTCTTACCTCAGTTACAATTTATA-3’) purchased from Gene Tools, LLC. DIG-RNA probes for whole-mount in situ hybridization were synthesized by in vitro transcription with T7 RNA polymerase as described previously [18]. Human recombinant bFGF (Sigma) and BMP4 (HumanZyme) were used at concentrations of 1 ng/mL and 100 ng/mL, respectively.

Identifiers for genes examined in the present study are as follows: CG.KH2012.C2.125 for *Fgf9/16/20*, CG.KH2012.C3.716 for *Efna*.*d*, CG.KH2012.C2.421 for *Admp*, CG.KH2012.C4.547 for *Gdf1/3-r*, and CG.KH2012.C4.84 for *Otx*.

## Supporting Information

S1 Fig Boolean functions in a hypothetical biological system, in which one signaling molecule is not freely diffusible. (A) A hypothetical biological system, consisting of two initially equivalent cells I and II, and two signaling molecules *a* and *b*. After a sufficient period of time, gene *o* is expressed only in cell I but not in cell II. Signaling molecule *a* is freely diffusible, while signaling molecule *b* is tethered to the cell membrane of its signaling source. (B) A Boolean function that describes expression of gene *o*. The 16 logically possible sensing patterns are shown in the second row. Because signaling molecule *b* is tethered to the cell membrane of its signaling source, signaling *b* is never transmitted to cell I. Hence, sensing patterns, 5–8 and 13–16, are incompatible and enclosed by boxes. Sensing patterns and Boolean functions incompatible with *Rules 1* and *2* (see text) are indicated by ‘X’ in the third row. The fourth and fifth rows show sensing patterns appearing in Experiments 1 and 2, which are shown in (C). Sensing patterns incompatible with *Rules 1* and *2* are shown in magenta in the second, fourth and fifth rows. Only sensing pattern 12 stands. The sixth row shows a Boolean function compatible with the sensing pattern among all of the logically possible Boolean functions shown in Fig 1D. (C) Two conceptual loss-of-function experiments. (D) A combination of Boolean functions and sensing patterns that explains the expression of gene *o* in this hypothetical system.
(PDF)

S2 Fig Boolean functions in a hypothetical biological system, in which one signaling molecule is not freely diffusible and expressed in induced cells. (A) A hypothetical biological system, consisting of two initially equivalent cells I and II, and two signaling molecules *a* and *b*. After a sufficient period of time, gene *o* is expressed only in cell I but not in cell II. Signaling molecule *a* is freely diffusible, while signaling molecule *b* is tethered to the cell membrane. Signaling molecule *b* is expressed in the signaling *b* source cell (shown in the bottom), cell I and cell II. (B) A Boolean function that describes expression of gene *o*. The 16 logically possible sensing patterns are shown in the second row. Sensing patterns and Boolean functions incompatible with *Rules 1* and *2* (see text) are indicated by ‘X’ in the third row. The fourth and fifth rows show sensing patterns appearing in Experiments 1 and 2, which are shown in (C). Sensing patterns incompatible with *Rules 1* and *2* are shown in magenta in the second, fourth and fifth rows. Only sensing pattern 12 stands. The sixth row shows a Boolean function compatible with the sensing pattern among all of the logically possible Boolean functions shown in Fig 1D. (C) Two conceptual loss-of-function experiments. (D) Two distinct combinations of Boolean functions and sensing patterns that explain the expression of gene *o* in this hypothetical system.
(PDF)

S3 Fig Sixteen Boolean functions that can explain *Otx* expression under normal conditions and in the experimental conditions published previously [3, 4]. Compatible sensing patterns (shown in Fig 3B) are shown in the bottom row. Outputs for four sensing patterns are not determined (magenta).
(PDF)

S4 Fig Sensing patterns and Boolean functions in three early 32-cell embryos. (A–C) Estimated order of signaling strength of the Admp, Efna.d, Fgf9/16/20 and Gdf1/3-r pathways for the ectodermal cells of the animal hemisphere of three early 32-cell virtually reconstructed embryos. (A), (B), and (C) show the order in the first, second, and third embryos that appear in S3 Table. (D, E) Four sensing patterns and eight Boolean functions can explain *Otx* expression in the normal and experimental conditions in the early 32-cell embryos. Sensing patterns 1, 2, and 3 are compatible with Boolean function A, whereas sensing pattern 4 is compatible with Boolean functions A to H, as shown in the bottom row of (D). Boolean functions A to H are shown in (E).
(PDF)

S1 Table Estimated contact areas of cells with surrounding cells expressing signaling ligands at the mid-to-late 32-cell stage. (PDF)

S2 Table Summary of expression of Otx in the animal hemisphere of the 32-cell embryo under normal and experimental conditions. (PDF)

S3 Table Estimated contact areas of cells with surrounding cells expressing signaling ligands in three different embryos at the early 32-cell stage. (PDF)
