# Supplementary figures and images for: A Boolean Function for Neural Induction Reveals a Critical Role of Direct Intercellular Interactions in Patterning the Ectoderm of the Ascidian Embryo
Source: PLoS Comput Biol. 2015 Dec 29;11(12):e1004687. doi: 10.1371/journal.pcbi.1004687 (PMC4695095; doi:10.1371/journal.pcbi.1004687)

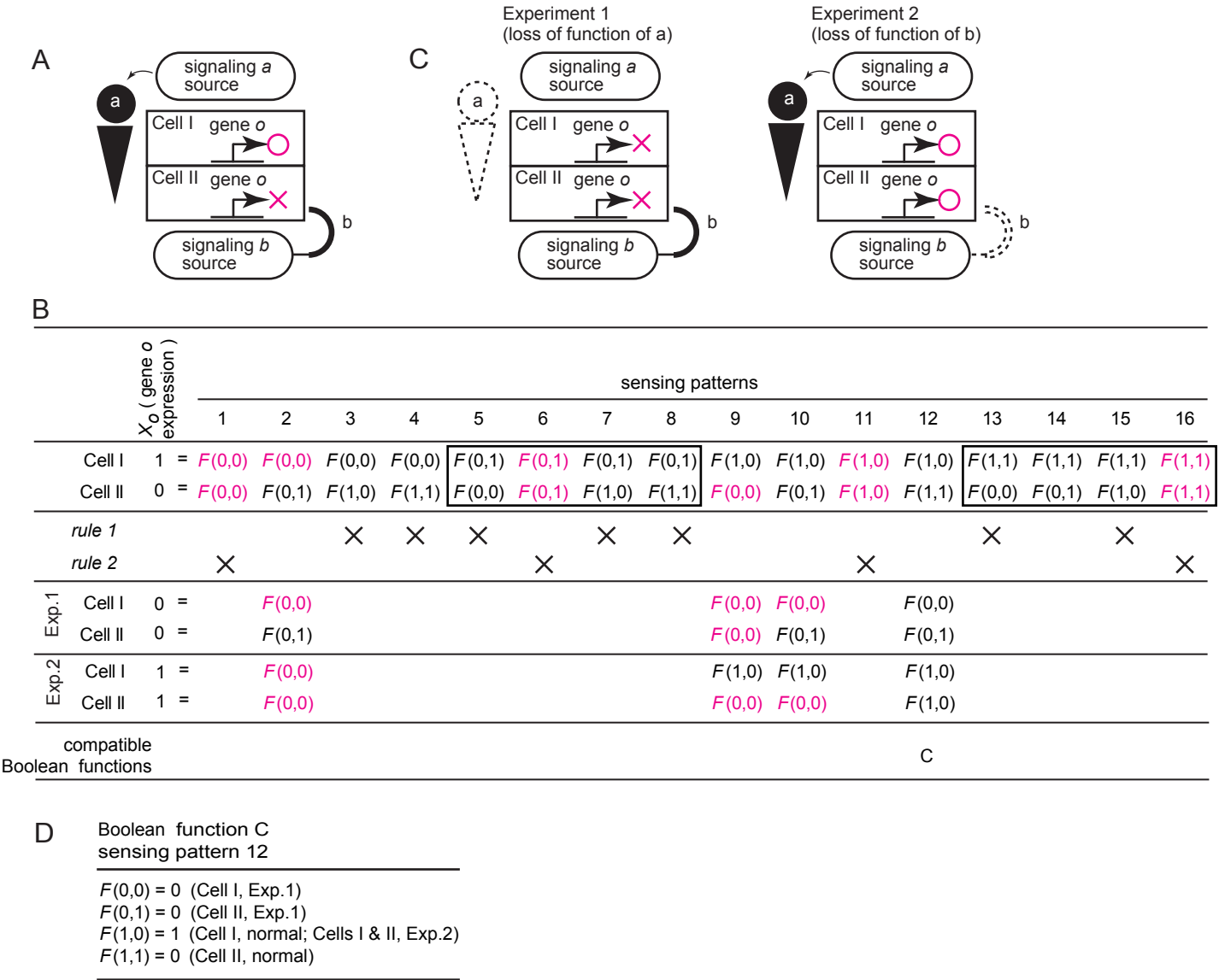

Supplement: S1 Fig — (A) A hypothetical biological system, consisting of two initially equivalent cells I and II, and two signaling molecules a and b. After a sufficient period of time, gene o is expressed only in cell I but not in cell II. Signaling molecule a is freely diffusible, while signaling molecule b is tethered to the cell membrane of its signaling source. (B) A Boolean function that describes expression of gene o. The 16 logically possible sensing patterns are shown in the second row. Because signaling molecule b is tethered to the cell membrane of its signaling source, signaling b is never transmitted to cell I. Hence, sensing patterns, 5–8 and 13–16, are incompatible and enclosed by boxes. Sensing patterns and Boolean functions incompatible with Rules 1 and 2 (see text) are indicated by ‘X’ in the third row. The fourth and fifth rows show sensing patterns appearing in Experiments 1 and 2, which are shown in (C). Sensing patterns incompatible with Rules 1 and 2 are shown in magenta in the second, fourth and fifth rows. Only sensing pattern 12 stands. The sixth row shows a Boolean function compatible with the sensing pattern among all of the logically possible Boolean functions shown in Fig 1D. (C) Two conceptual loss-of-function experiments. (D) A combination of Boolean functions and sensing patterns that explains the expression of gene o in this hypothetical system. (PDF) [file pcbi.1004687.s001.pdf]

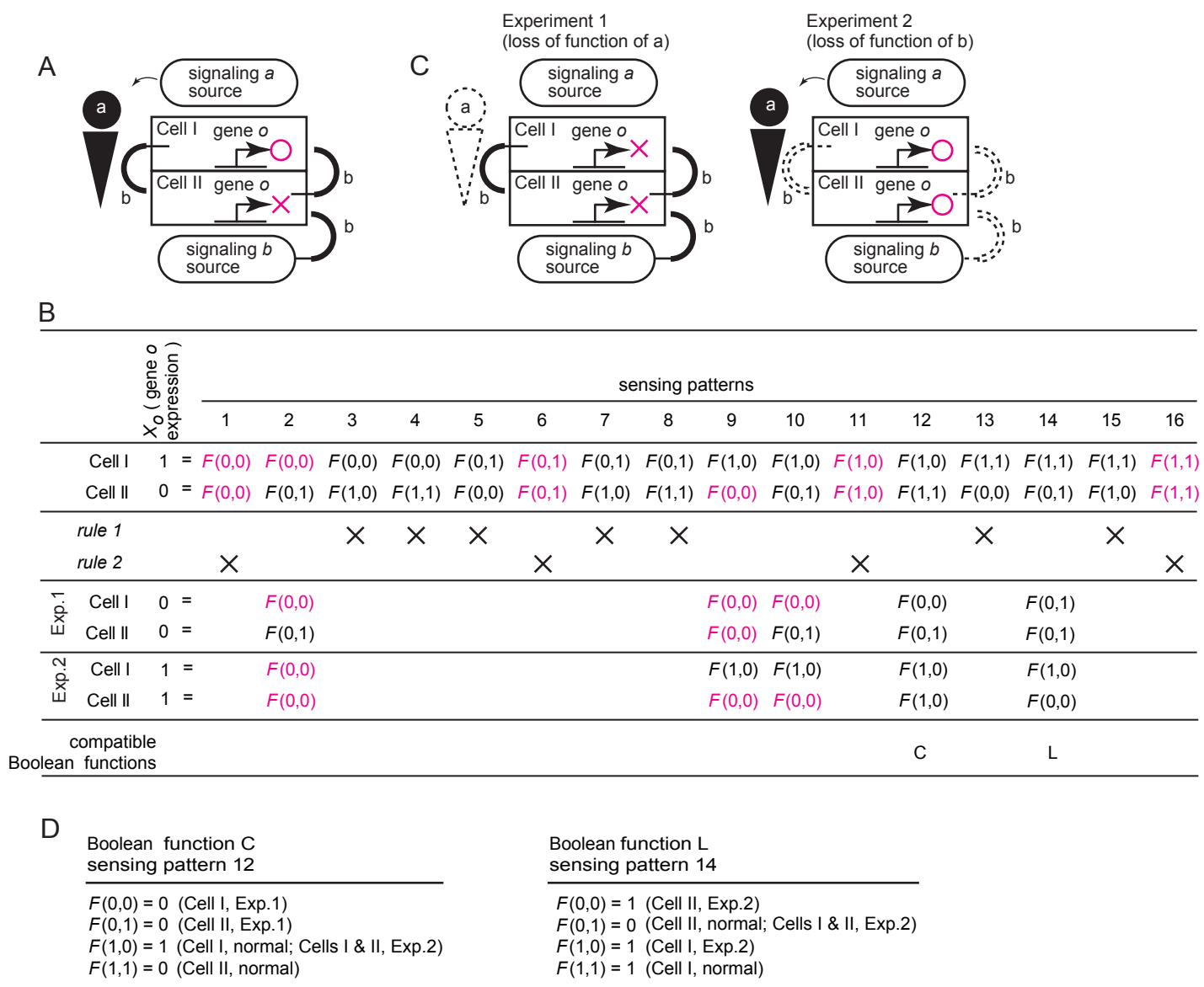

Supplement: S2 Fig — (A) A hypothetical biological system, consisting of two initially equivalent cells I and II, and two signaling molecules a and b. After a sufficient period of time, gene o is expressed only in cell I but not in cell II. Signaling molecule a is freely diffusible, while signaling molecule b is tethered to the cell membrane. Signaling molecule b is expressed in the signaling b source cell (shown in the bottom), cell I and cell II. (B) A Boolean function that describes expression of gene o. The 16 logically possible sensing patterns are shown in the second row. Sensing patterns and Boolean functions incompatible with Rules 1 and 2 (see text) are indicated by ‘X’ in the third row. The fourth and fifth rows show sensing patterns appearing in Experiments 1 and 2, which are shown in (C). Sensing patterns incompatible with Rules 1 and 2 are shown in magenta in the second, fourth and fifth rows. Only sensing pattern 12 stands. The sixth row shows a Boolean function compatible with the sensing pattern among all of the logically possible Boolean functions shown in Fig 1D. (C) Two conceptual loss-of-function experiments. (D) Two distinct combinations of Boolean functions and sensing patterns that explain the expression of gene o in this hypothetical system. (PDF) [file pcbi.1004687.s002.pdf]
