# Supplementary material for: A Boolean Function for Neural Induction Reveals a Critical Role of Direct Intercellular Interactions in Patterning the Ectoderm of the Ascidian Embryo
Source: PLoS Comput Biol. 2015 Dec 29;11(12):e1004687. doi: 10.1371/journal.pcbi.1004687 (PMC4695095; doi:10.1371/journal.pcbi.1004687)
Supplement: S3 Fig — Compatible sensing patterns (shown in Fig 3B) are shown in the bottom row. Outputs for four sensing patterns are not determined (magenta). (PDF) [file pcbi.1004687.s003.pdf]

$F(X_{admp}, X_{efn}, X_{fgf}, X_{gdf})$	function	*Otx* expression (X_o)															
		A	B	C	D	E	F	G	H	I	J	K	L	M	N	O	P
F (0,0,0,0)	=	0	0	0	0	0	0	0	0	0	0	0	0	0	0	0	0
F (0,0,0,1)	=	0	0	0	0	0	0	0	0	1	1	1	1	1	1	1	1
F (0,0,1,0)	=	1	1	1	1	1	1	1	1	1	1	1	1	1	1	1	1
F (0,0,1,1)	=	1	1	1	1	1	1	1	1	1	1	1	1	1	1	1	1
F (0,1,0,0)	=	0	0	0	0	0	0	0	0	0	0	0	0	0	0	0	0
F (0,1,0,1)	=	0	0	0	0	1	1	1	1	0	0	0	0	1	1	1	1
F (0,1,1,0)	=	1	1	1	1	1	1	1	1	1	1	1	1	1	1	1	1
F (0,1,1,1)	=	0	0	0	0	0	0	0	0	0	0	0	0	0	0	0	0
F (1,0,0,0)	=	0	0	1	1	0	0	1	1	0	0	1	1	0	0	1	1
F (1,0,0,1)	=	0	0	0	0	0	0	0	0	0	0	0	0	0	0	0	0
F (1,0,1,0)	=	1	1	1	1	1	1	1	1	1	1	1	1	1	1	1	1
F (1,0,1,1)	=	1	1	1	1	1	1	1	1	1	1	1	1	1	1	1	1
F (1,1,0,0)	=	0	1	0	1	0	1	0	1	0	1	0	1	0	1	0	1
F (1,1,0,1)	=	0	0	0	0	0	0	0	0	0	0	0	0	0	0	0	0
F (1,1,1,0)	=	0	0	0	0	0	0	0	0	0	0	0	0	0	0	0	0
F (1,1,1,1)	=	0	0	0	0	0	0	0	0	0	0	0	0	0	0	0	0
Compatible Sensing patterns		1<br>2<br>3	1<br>2<br>3	1<br>3	1<br>3	1<br>2<br>3	1<br>2<br>3	1<br>3	1<br>3	1<br>2	1<br>2	1	1	1<br>2	1<br>2	1	1
