# Supplementary material for: A Boolean Function for Neural Induction Reveals a Critical Role of Direct Intercellular Interactions in Patterning the Ectoderm of the Ascidian Embryo
Source: PLoS Comput Biol. 2015 Dec 29;11(12):e1004687. doi: 10.1371/journal.pcbi.1004687 (PMC4695095; doi:10.1371/journal.pcbi.1004687)
Supplement: S4 Fig — (A–C) Estimated order of signaling strength of the Admp, Efna.d, Fgf9/16/20 and Gdf1/3-r pathways for the ectodermal cells of the animal hemisphere of three early 32-cell virtually reconstructed embryos. (A), (B), and (C) show the order in the first, second, and third embryos that appear in S3 Table. (D, E) Four sensing patterns and eight Boolean functions can explain Otx expression in the normal and experimental conditions in the early 32-cell embryos. Sensing patterns 1, 2, and 3 are compatible with Boolean function A, whereas sensing pattern 4 is compatible with Boolean functions A to H, as shown in the bottom row of (D). Boolean functions A to H are shown in (E). (PDF) [file pcbi.1004687.s004.pdf]

Ohta et al., S4 Figure

A

Admp	a6.5 ≤ a6.7 ≤ a6.6 ≤ a6.8 ≤ b6.8 ≤ b6.7 ≤ b6.6 ≤ b6.5
EfnA.d	b6.5 ≤ a6.5 ≤ b6.7 ≤ a6.7 ≤ b6.6 ≤ a6.6 ≤ a6.8 ≤ b6.8
Fgf9/16/20	a6.8 ≤ b6.8 ≤ a6.6 ≤ b6.7 ≤ a6.7 ≤ b6.6 ≤ a6.5 ≤ b6.5
Gdf1/3-r	a6.5 ≤ b6.5 ≤ a6.7 ≤ b6.7 ≤ b6.6 ≤ a6.6 ≤ a6.8 ≤ b6.8

B

Admp	a6.5 ≤ a6.7 ≤ a6.6 ≤ a6.8 ≤ b6.8 ≤ b6.7 ≤ b6.6 ≤ b6.5
EfnA.d	b6.5 ≤ a6.5 ≤ b6.7 ≤ a6.7 ≤ b6.6 ≤ a6.6 ≤ b6.8 ≤ a6.8
Fgf9/16/20	a6.8 ≤ b6.7 ≤ b6.8 ≤ a6.6 ≤ a6.7 ≤ b6.6 ≤ a6.5 ≤ b6.5
Gdf1/3-r	a6.5 ≤ b6.5 ≤ a6.7 ≤ b6.7 ≤ b6.6 ≤ a6.6 ≤ a6.8 ≤ b6.8

C

Admp	a6.5 ≤ a6.7 ≤ a6.6 ≤ a6.8 ≤ b6.8 ≤ b6.7 ≤ b6.5 ≤ b6.6
EfnA.d	b6.5 ≤ a6.5 ≤ b6.7 ≤ a6.7 ≤ b6.6 ≤ a6.6 ≤ a6.8 ≤ b6.8
Fgf9/16/20	a6.8 ≤ a6.6 ≤ b6.8 ≤ a6.7 ≤ b6.7 ≤ b6.6 ≤ a6.5 ≤ b6.5
Gdf1/3-r	a6.5 ≤ b6.5 ≤ a6.7 ≤ b6.7 ≤ b6.6 ≤ a6.6 ≤ a6.8 ≤ b6.8

D

		sensing pattern			
Cell	(*Xo*) *Otx* expression	1	2	3	4
a6.5	1 =	*F* (1,0,1,1)	*F* (1,0,1,0)	*F* (0,0,1,1)	*F* (1,0,1,0)
a6.6	0 =	*F* (1,1,1,1)	*F* (1,1,1,1)	*F* (1,1,1,1)	*F* (1,1,1,1)
a6.7	0 =	*F* (1,1,1,1)	*F* (1,1,1,1)	*F* (1,1,1,1)	*F* (1,1,1,1)
a6.8	0 =	*F* (1,1,1,1)	*F* (1,1,1,1)	*F* (1,1,1,1)	*F* (1,1,1,1)
b6.5	1 =	*F* (1,0,1,1)	*F* (1,0,1,1)	*F* (1,0,1,1)	*F* (1,0,1,0)
b6.6	0 =	*F* (1,1,1,1)	*F* (1,1,1,1)	*F* (1,1,1,1)	*F* (1,1,1,1)
b6.7	0 =	*F* (1,1,1,1)	*F* (1,1,1,1)	*F* (1,1,1,1)	*F* (1,1,1,1)
b6.8	0 =	*F* (1,1,1,1)	*F* (1,1,1,1)	*F* (1,1,1,1)	*F* (1,1,1,1)
compatible logic functions		A	A	A	A to H

E

$F(X_{admp}, X_{efn}, X_{fgf}, X_{gdf})$	function	*Otx* expression (X_o) A	B	C	D	E	F	G	H
F (0,0,0,0)	=	0	0	0	0	0	0	0	0
F (0,0,0,1)	=	0	0	0	0	1	1	1	1
F (0,0,1,0)	=	1	1	1	1	1	1	1	1
F (0,0,1,1)	=	1	0	0	1	0	0	1	1
F (0,1,0,0)	=	0	0	0	0	0	0	0	0
F (0,1,0,1)	=	0	0	0	0	0	0	0	0
F (0,1,1,0)	=	1	1	1	1	1	1	1	1
F (0,1,1,1)	=	0	0	0	0	0	0	0	0
F (1,0,0,0)	=	0	0	0	0	0	0	0	0
F (1,0,0,1)	=	0	0	1	1	0	1	0	1
F (1,0,1,0)	=	1	1	1	1	1	1	1	1
F (1,0,1,1)	=	1	1	1	1	1	1	1	1
F (1,1,0,0)	=	0	0	0	0	0	0	0	0
F (1,1,0,1)	=	0	0	0	0	0	0	0	0
F (1,1,1,0)	=	0	0	0	0	0	0	0	0
F (1,1,1,1)	=	0	0	0	0	0	0	0	0
