# Supplementary material for: A Boolean Function for Neural Induction Reveals a Critical Role of Direct Intercellular Interactions in Patterning the Ectoderm of the Ascidian Embryo
Source: PLoS Comput Biol. 2015 Dec 29;11(12):e1004687. doi: 10.1371/journal.pcbi.1004687 (PMC4695095; doi:10.1371/journal.pcbi.1004687)
Supplement: S1 Table — (PDF) [file pcbi.1004687.s005.pdf]

S1 Table. Estimated contact areas of cells with surrounding cells expressing signaling ligands at the mid-to-late 32-cell stage.

		Contact area (µm^2)[*1]				
Stage	Cell	Admp	EfnA.d	Fgf9/16/20	Gdf1/3-r	Embryo data[*3]
Mid-to-Late	a6.5	(424)[*2]	5,437	4,462	15,336	Mid_Late_32-cell_stage_Bitplane_1.txt
	a6.6	(278)[*2]	11,374	1,332	24,080	
	a6.7	(347)[*2]	6,788	2,648	16,224	
	a6.8	(163)[*2]	11,730	1,206	24,666	
	b6.5	2,566	4,213	8,273	16,699	
	b6.6	3,032	10,089	3,456	23,634	
	b6.7	2,184	7,054	2,184	19,947	
	b6.8	883	13,503	964	28,026	

[*1] Values in this table were calculated using data for areas of contact between blastomeres from Tassy et al., 2006 [7], and gene expression data from Bertrand et al., 2003 [3], and Imai et al., 2004 [18].

[*2] Total distance from cells expressing Admp (µm).

[*3] File name appearing in the Aniseed database.
