# Supplementary material for: A Boolean Function for Neural Induction Reveals a Critical Role of Direct Intercellular Interactions in Patterning the Ectoderm of the Ascidian Embryo
Source: PLoS Comput Biol. 2015 Dec 29;11(12):e1004687. doi: 10.1371/journal.pcbi.1004687 (PMC4695095; doi:10.1371/journal.pcbi.1004687)
Supplement: S2 Table — (PDF) [file pcbi.1004687.s006.pdf]

S2 Table. Summary of expression of *Otx* in the animal hemisphere of the 32-cell embryo under normal and experimental conditions.

Cell	Control	*Admp* MO	*EfnA.d* MO	*Fgf9/16/20* MO	*Gdf1/3-r* MO	*Admp* & *Gdf1/3-r* MO	*Admp* & *Fgf9/16/20* & *Gdf1/3-r* MO	*Admp* RNA	*EfnA.d* RNA	*Gdf1/3-r* RNA
a6.5	1	1	1	0	1	1	0	1	0	1
a6.6	0	0	1	0	0	1	0	0	0	0
a6.7	0	0	1	0	0	1	0	0	0	0
a6.8	0	0	1	0	0	1	0	0	0	0
b6.5	1	1	1	0	1	1	0	1	0	1
b6.6	0	0	1	0	0	1	0	0	0	0
b6.7	0	0	1	0	0	1	0	0	0	0
b6.8	0	0	1	0	0	1	0	0	0	0

Cells expressing *Otx* are indicated by ‘1’, and cells not expressing *Otx* are indicated by ‘0’. This table is based on data published in our previous study [4].
