# Supplementary material for: A Boolean Function for Neural Induction Reveals a Critical Role of Direct Intercellular Interactions in Patterning the Ectoderm of the Ascidian Embryo
Source: PLoS Comput Biol. 2015 Dec 29;11(12):e1004687. doi: 10.1371/journal.pcbi.1004687 (PMC4695095; doi:10.1371/journal.pcbi.1004687)
Supplement: S3 Table — (PDF) [file pcbi.1004687.s007.pdf]

S3 Table. Estimated contact areas of cells with surrounding cells expressing signaling ligands in three different embryos at the early 32-cell stage.

		Contact area (μm^2)*1				
Stage	Cell	Admp	EfnA.d	Fgf9/16/20	Gdf1/3-r	Embryo data*3
Early*4	a6.5	(353)*2	6,370	5,510	18,250	Early_32-cell_stage_Amira_High-Resolution_4.txt
	a6.6	(170)*2	15,460	2,513	33,433	
	a6.7	(231)*2	10,725	3,504	24,954	
	a6.8	(97)*2	18,852	1,344	39,048	
	b6.5	4,226	4,742	10,976	20,460	
	b6.6	3,703	12,977	4,513	30,467	
	b6.7	2,908	9,266	2,908	26,560	
	b6.8	2,080	19,784	2,471	42,039	
Early	a6.5	(336)*2	5,823	4,767	16,413	Early_32-cell_stage_Amira_High-Resolution_5.txt
	a6.6	(137)*2	13,552	2,605	29,709	
	a6.7	(244)*2	8,956	2,947	20,859	
	a6.8	(86)*2	16,691	1,395	34,777	
	b6.5	3,412	3,997	9,259	17,253	
	b6.6	3,141	11,237	4,177	26,651	
	b6.7	2,380	7,432	2,380	22,305	
	b6.8	2,150	16,684	2,589	35,991	
Early	a6.5	(291)*2	6,934	5,148	19,016	Early_32-cell_stage_Amira_High-Resolution_6.txt
	a6.6	(112)*2	15,319	2,058	32,696	
	a6.7	(216)*2	10,467	2,838	23,772	
	a6.8	43	19,056	865	38,977	
	b6.5	3,664	4,674	10,240	19,588	
	b6.6	3,740	13,168	4,674	31,010	
	b6.7	3,018	9,273	3,018	26,265	
	b6.8	2,121	19,469	2,324	41,262	

*1 Values in this table were calculated using data for areas of contact between blastomeres from Tassy et al., 2006 [7], and gene expression data from Bertrand et al., 2003 [3], and Imai et al., 2004 [18].

*2 Total distance from cells expressing Admp (μm).

*3 File name appeared in the Aniseed database [22].

*4 The contact surfaces for *Fgf9/16/20* and *EfnA.d* are identical to those in our previous study [4].
